# Latent tuberculosis infection and associated risk factors among people living with HIV and apparently healthy blood donors at the University of Gondar referral hospital, Northwest Ethiopia

**DOI:** 10.1186/s13104-019-4548-x

**Published:** 2019-08-16

**Authors:** Mekdes Tilahun, Agumas Shibabaw, Amare Kiflie, Gezahegn Bewket, Ebba Abate, Baye Gelaw

**Affiliations:** 1 Department of Medical Microbiology, School of Biomedical and Laboratory Sciences, College of Medicine and Health Sciences (CMHS), The University of Gondar (UOG), P.O. box 196, Gondar, Ethiopia; 2 Department of Immunology and Molecular Biology, School of Biomedical and Laboratory Sciences, College of Medicine and Health Sciences (CMHS), The University of Gondar (UOG), P.O. box 196, Gondar, Ethiopia; 3 Ethiopian Public Health Institute (EPHI), P.O. box 1242, Addis Ababa, Ethiopia

**Keywords:** Tuberculosis, Latent TB, HIV, Blood donor, QFT-GIT

## Abstract

**Objective:**

Immuno-compromised individuals with latent tuberculosis infection (LTBI) are at an increased risk for tuberculosis reactivation compared with the general population. The aim of this study was to determine the prevalence of latent tuberculosis infection among people living with human immunodeficiency virus (PLWH) and apparently healthy blood donors. Human Immunodeficiency Virus positive individuals and for the purpose of comparison apparently healthy blood donors were enrolled. Blood sample was collected and tested for LTBI using QuantiFeron-TB Gold In-Tube assay (QFT-GIT) and CD4+ T cell count was determined by using BD FACS count.

**Results:**

The overall prevalence of LTBI regardless of HIV status was 46%. The prevalence of LTBI among PLWH was 44% and that of blood donors 48%. ART naïve HIV positive patients were three times more likely to have LTBI than patients under ART treatment (P = 0.04). Data also showed statistically significant negative association between previous or current preventive INH therapy and LTBI among HIV positive cases (P = 0.005). The proportion of LTBI was slightly lower among HIV positive individuals than apparently healthy blood donors. Nevertheless, HIV positive individuals should be screened for LTBI and take INH prophylaxis.

## Introduction

The risk for developing active TB among HIV positive individuals increased many folds even during anti-HIV treatment [1]. Moreover, HIV infection is among the most important risk factors for the progression of latent tuberculosis infection to active TB [2]. Latent tuberculosis infection (LTBI) is characterized by the presence of immune responses to previously acquired *Mycobacterium tuberculosis* infection without clinical evidence of active tuberculosis (TB). Persons with LTBI are not infectious and cannot transmit TB infection to others and have negative sputum tests [3].

The prevalence of LTBI has increased worldwide with marked variations in different regions. It is generally high in developing countries than the developed ones [4, 5]. The prevalence of LTBI among blood specimen collected from HIV patients in Italy was found 9.5% [6]. High LTBI (68.1%) among HIV positive cases was reported in Spain [4]. In Africa, higher prevalence of LTBI (69% and 76%) among HIV positive cases was reported in South Africa [7] and Tanzania [8], respectively. The prevalence of LTBI among apparently healthy adults and healthy blood donors in Afar region (north-east Ethiopia) and Gondar (north-west Ethiopia) was reported as 31.2% and 51%, respectively [9].

Latent tuberculosis infection (LTBI) in immuno-compromised individuals may progress to severe active tuberculosis (TB) disease and serve as a reservoir for future transmission of TB disease [10]. The identification and successful treatment of individuals with LTBI is important for the fundamental understanding of the pathogenesis of the disease, support ongoing efforts to develop new TB vaccines, and reduce the subsequent risk for the re-activation and development of active TB [11]. Currently, LTBI screening is recommended for target populations such as patients receiving tumor necrosis factor treatment, cases co-infected with HIV and children aged less than 5 years who are at high risk of developing tuberculosis. Meanwhile, prophylaxis treatment is an alternative for tuberculosis control for high risk populations [12].

Immuno-compromised persons with latent tuberculosis infection are at increased risk for tuberculosis reactivation compared with the general population [13]. Therefore, it is important to determine the prevalence of LTBI among individuals who are at higher risk of developing active tuberculosis. However, the burden of latent tuberculosis among PLWH compared to healthy blood donors is not fully understood in Ethiopia. Therefore, the aim of this study was to determine the prevalence of LTBI and associated risk factors among HIV positive individuals and healthy blood donors.

## Main text

### Materials and methods

#### Study area, design and period

The study was conducted at the University of Gondar referral hospital located in Gondar town, one of the ancient and densely populated towns in Ethiopia. According to the 2015 population and housing census result of Ethiopia, the town had total population of 206,987.

#### Populations

The source population was all patients seeking health service at the University of Gondar referral hospital during the study period. The study populations were individuals living with HIV and apparently healthy blood donors who visited the University of Gondar referral hospital for health service and blood donation, respectively, from February 2016 to May 2017.

#### Inclusion and exclusion criteria

HIV positive individuals with CD4+ T cell count ≥ 200 cells/mm^3^ and apparently healthy blood donors were included in the study. HIV positive individuals who had CD4+ T cell count less than 200 cells/mm^3^ and individuals that had active TB infection were excluded from the study.

#### Study variables

The prevalence of latent tuberculosis infection was used as the dependent variable, while socio-demographic characteristics, BMI, TB contact, BCG vaccination, smoking habit, CD4+ cell count, family history of TB, previous uses of INH prophylaxis, current use of INH prophylaxis, ART status and duration of ART therapy were used as the independent variables.

#### Sample size determination and sampling technique

Sample size was determined by using a 50% prevalence of LTBI. We used a 20% (P1) and a 30% (P2) prevalence of LTBI among HIV+ patients and blood donors, respectively. At a 95% confidence interval and 80% power of test, n_1_ versus n_2_ 1:1 ratio the initial sample size was determined 206. Considering a 10% non-response rate, the final sample size was 226 (113 HIV positive and 113 healthy controls).

#### Sampling techniques

Study participants were selected by using the systematic random sampling. Data of the Anti-Retroviral Treatment (ART) clinic showed that 1800 patients visited the clinic per month. Data collection period for this study was planned for 3 months and 3 × 1800 = 5400: k = N/n, 5400/113 = 48. Then, the first individual was selected randomly from patients registered 1 to 48 and included in the study. Then, every 48th individual who visited the ART clinic was selected and included in the study until the required numbers of study subjects were obtained. The required number of individuals among the apparently healthy individuals was selected by using the systematic random sampling method, too. Nearly 30 clients visited the Gondar Red Cross blood bank center every day which equals 30 × 30 × 3 = 2700, and 2700/113 = 24. Therefore, every 24th apparently healthy blood donor was recruited and included in the study, and the 1st client was selected randomly from blood donors registered 1 to 24.

#### Socio-demographic and clinical data collection

Socio-demographic characteristics of participants were collected using a pretested and structured questionnaire prepared in English and translated into the local language, Amharic. The prepared questionnaire was checked for completeness and validity prior to the collection of data at Gondar Poly clinic.

#### Sample collection and processing

Blood sample was collected by strictly following the standard operational procedures of the University of Gondar hospital laboratory. Six milliliter of venous blood was collected and 3 ml deposited into a tube containing EDTA anticoagulant and QFT-GIT test tubes. Following the standard operational procedures of the University of Gondar hospital laboratory, the blood samples were tested for quantiferon (QFT-GIT test) and CD4+ T cell count.

#### QuantiFERON-TB Gold In-Tube assay (QFN-GIT)

One milliliter of blood sample was transferred to each of the three QFT-GIT test blood collection tubes (a phytohemagglutinin, positive control (Mitogen) and a negative (Nil) control tube). Interferon-γ concentration was determined from the whole-blood supernatant by ELISA following the manufacturers’ description.

#### CD4+ lymphocyte count

CD4+ T cell count was conducted by incubating anti-coagulated whole blood with monoclonal antibodies. The specimens were analyzed on a flowcytometer to determine the proportion of cells of a particular phenotype. Tests were performed based on the manufacturer’s instructions.

#### Data analysis and interpretation

Data was checked for completeness, cleaned manually and entered into Epi Info Version7 and analyzed using SPSS version 20 computer software. P-values less than 0.05 were considered as statistically significant.

### Results and discussion

Latent tuberculosis infection is a state of persistent immune response to stimulation by *Mycobacterium tuberculosis* antigens without evidence of clinically a manifested active TB [14]. HIV infection has contributed to a significant increase in the worldwide incidence of TB [15]. Because of the underlying immune deficiency, HIV-infected individuals with LTBI are at 26-fold higher risk for TB reactivation [16]. There are study reports that highlighted the importance of baseline LTBI screening for all HIV patients for minimizing the occurrence of clinical TB diseases and reducing TB incidence in the population [17].

#### Socio demographic characteristics

Almost half of the participants were females (53.5%). About 63% of the HIV+ cases were females but 56% of the apparently health blood donors were males. The mean ages of the HIV cases and apparently healthy blood donors were 37 and 28 years, respectively. Data also showed that 92% of the HIV positive cases and 85.8% of the apparently healthy blood donors were urban dwellers (Table 1).

**Table 1:** Socio demographic characteristics and risk factors of PLWH and apparently healthy blood donors at Gondar University referral hospital, Northwest Ethiopia, 2017

Characteristics	PLWH n (%)	Blood donor’s n (%)	Total n (%)
Mean (± SD) Age	36.9 (± 6.6)	27.9 (± 5.5)	32.4 (± 7.5)
Age (years)			
< 30	16 (14.2)	73 (64.6)	89 (39.4)
30–39	57 (50.4)	38 (33.6)	95 (42.0)
> 39	40 (35.4)	2 (1.8)	42 (18.6)
Sex			
Male	42 (37.2)	63 (55.8)	105 (46.5)
Female	71 (62.8)	50 (44.2)	121 (53.5)
Educational status			
Unable to read and write	20 (17.7)	5 (4.4)	25 (11.1)
Primary	57 (50.4)	24 (21.2)	81 (35.8)
Secondary	21 (18.6)	43 (38.1)	64 (28.3)
Collage and above	15 (13.3)	41 (36.3)	56 (24.8)
Occupation			
House wife	37 (32.7)	8 (7.1)	45 (19.9)
Farmer	5 (4.4)	5 (7.1)	12 (5.3)
Merchant	29 (25.7)	19 (16.8)	47 (20.8)
Employee	29 (25.7)	52 (46.0)	81 (35.8)
Student	2 (1.8)	25 (22.1)	27 (11.9)
Daily labor	11 (9.7)	4 (3.5)	14 (6.2)
Religion			
Orthodox	66 (58.4)	99 (87.6)	165 (73)
Muslim	28 (24.8)	8 (7.1)	36 (15.9)
Protestant	19 (16.8)	6 (5.3)	25 (11.1)
Ethnicity			
Amhara	104 (92)	103 (87.6)	207 (92.5)
Oromo	6 (5.3)	5 (4.4)	11 (4.4)
Tigrie	3 (2.7)	5 (4.4)	8 (3.1)
Monthly income			
< 800	43 (38.1)	15 (13.3)	58 (25.7)
800–1500	29 (25.7)	33 (29.2)	62 (27.4)
1501–3025	26 (23.0)	24 (21.2)	50 (22.1)
> 3025	15 (13.3)	41 (36.3)	56 (24.8)

#### Co-morbidities among PLWH and blood donors

Tuberculosis contact was found on 37.2% of the HIV positive cases and 31% of the blood donors. Close contact with pulmonary tuberculosis positive cases is a risk factor for acquisition of LTBI [18]. Marks et al. reported that the prevalence of LTBI was about 36% in close contacts of persons with infectious TB as assessed by TST in a study conducted in Iran [19]. INH prophylaxis was given to 25.7% of the HIV positive cases and 64.1% of them had ART for a duration of 5–10 years; 58.4% of the HIV positive individuals had CD4 count < 500 cells/mm^3^. On the other hand, only 3.5% of the apparently healthy blood donors had CD4+ T cell count < 500 cells.

#### Prevalence of latent tuberculosis infection

The overall prevalence of LTBI was 46.0%, comparable with the prevalence of LTBI reported previously in Uganda (49%) and Zambia (40%) [15, 20]. The prevalence of LTBI among HIV positive cases was 44.2%, while that of the apparently healthy blood donors was 47.8%. The prevalence of LTBI among PLWH found in the current study was by far higher than those of studies conducted in Saudi Arabia (5.0%) [21], Atlanta (8.0%) [22], Norway and the UK (10%) [23]. A 51% prevalence of LTBI was reported among blood donors in the same study area previously [24].

**Fig. 1:**
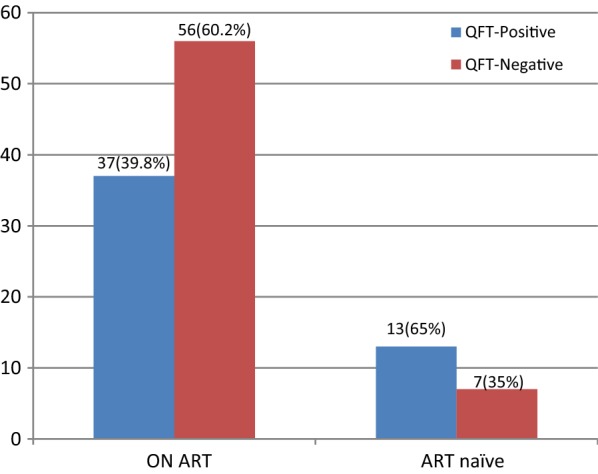
Prevalence of LTBI among HIV positive cases who are taking ART compared with ART naïve patients at the University of Gondar referral hospital, Northwest Ethiopia, 2017

Among the HIV positive cases, 93 were on anti-retroviral therapy; the other 20 were treatment naïve. The prevalence of LTBI among HIV positive cases taking ART and ART naïve groups was 39.8% and 65%, respectively (Fig. 1). Moreover, ART naïve HIV positive patients were 3 times more likely to have LTBI than patients under ART treatment [P = 0.04; COR (95% CI) 2.8 (1.02–7.7)]. It has previously been shown that antiretroviral therapy decreases the incidence of tuberculosis among HIV-infected persons [25–27]. The reduction in TB disease with antiretroviral therapy may be due to an improved ability to control LTBI. Antiretroviral therapy has also been associated with lower TB recurrence rates and the decrease of mortality [28].

#### Association of risk factors with LTBI among PLWH and blood donors

Among PLWH, the majority (52.5%) of the LTBI individuals were found within 30–39 years of age, but 62.5% of the blood donors that had LTBI were under 30 years of age. Previously, Lee et al. reported that LTBI prevalence increased with age. The risk of infection from household and community sources increased from birth until 20 years of age [29]. Among PLWH slightly over half (50.7%) of the LTBI positive individuals were females. In fact, a higher prevalence of LTBI (43% versus 33%) was reported among males compared to females [30]. In both cases, the majority had BMI greater than 17 kg/m^2^. On the other hand, PLWH and apparently healthy blood donors who had close contacts with TB patient were about 5 and 4 times more likely to develop LTBI as compared to individuals who had no close contacts with TB patients [P = 0.003, AOR = 5.2 (95% CI 1.7–15.8) and P = 0.003, AOR = 4.5 (95% CI 1.7–11.9)], respectively. Similarly, PLWH and apparently healthy blood donors who had family history of TB infection had about 4 times higher association to LTBI compared to those who did not have such history [P = 0.023, AOR = 3.8 (95% CI 1.2–12 and P = 0.025, AOR = 3.9 (95% CI 1.2–13), respectively] (Table 2). Cheng et al. previously reported that close contact with tuberculosis was a risk factor for LTBI [31].

**Table 2:** Association between the prevalence of LTBI and risk factors among PLWH at University of Gondar referral hospital, Northwest Ethiopia, 2017

Characteristics	QuantiFERON test result					
	Positive n (%)	Negative N (%)	P-value	COR (95% C.I.)	P-value	AOR (95% C.I.)
Contact with TB case						
Yes	31 (73.8)	11 (26.2)	0.0	7.71 (3.2–18.3)	0.003*	5.2 (1.7–15.8)*
No	19 (26.8)	52 (73.2)	–	1.0	–	1.0
Family history with TB						
Yes	26 (74.3)	9 (25.7)	0.0	6.5 (2.6–16)	0.023*	3.8 (1.2–12)*
No	24 (30.8)	54 (69.2)	–	1.0	–	3.79 (1.0)
INH prophylaxis						
Yes	4 (13.8)	25 (86.2)	0.0	0.1 (0.1–0.4)	0.005*	0.1 (0.03–0.6)*
No	46 (54.8)	38 (45.2)	–	1.0	–	1.0
ART status						
Yes	37 (39.8)	56 (60.0)	–	1.00	–	–
No	13 (65.0)	7 (35.0)	0.04	2.8 (1–7.7)	0.138	2.6 (0.7–9.1)

*ART* antiretroviral therapy, *HIV* human immunodeficiency virus, *INH* isonicotinylhydrazide (Isoniazid)

* P-value = < 0.05

### Conclusion

The overall prevalence of LTBI was 46%, and the prevalence of LTBI among PLWH was slightly lower than that of the blood donors. Close contacts with known TB cases and family history of TB were found risk factors for LTBI. Therefore, screening all HIV positive cases for LTBI could be valuable to initiate tuberculosis treatment and to prevent its progress to active tuberculosis infection.

## Limitations

In this study, population groups less than 18 years of age were not included. Because the study design was cross-sectional, the exact relationship to develop LTBI among HIV patients which could have been assessed in a cohort study was not determined, and that was the limitation of the attempt.

## Data Availability

Data were registered on Microsoft excel spread sheet and the datasets are available from the correspondence author but will not be shared to ensure patient confidentiality.

## Abbreviations

AIDS: Acquired Immunodeficiency Syndrome
ART: anti retroviral therapy
CD4: cluster of differentiation 4
ELISA: Enzyme Linked Immuno Sorbent Assay
ESAT-6: early secreted antigenic target 6
HIV: human immunodeficiency virus
IFN: interferon
IGRA: interferon gamma release assay
INH: isonicotinylhydrazide
IPT: isoniazid preventive therapy
LTBI: latent tuberculosis infection
MTB: *Mycobacterium tuberculosis*
PLWH: people living with HIV
PPD: purified protein derivative
QFT-GIT: QuantiFERON-TB Gold In-Tube method
TB: tuberculosis
TST: tuberculin skin test
WHO: World Health Organization
